# Seasonality in the dung beetle community in a Brazilian tropical dry forest: Do small changes make a difference?

**DOI:** 10.1093/jis/14.1.123

**Published:** 2014-09-01

**Authors:** Anderson Matos Medina, Priscila Paixão Lopes

**Affiliations:** 1 Postgraduate Program in Zoology (Programa de Pos-Graduacao em Zoologia), State University of Feira de Santana, (Universidade Estadual de Feira de Santana: UEFS), Av. Transnordestina s/n, 44036-900, Feira de Santana, Bahia, Brazil; 2 Department of Biological Sciences, (Departamento de Ciencias Biologicas) State University of Feira de Santana, (Universidade Estadual de Feira de Santana: UEFS), Av. Transnordestina s/n, 44036-900, Feira de Santana, Bahia, Brazil

**Keywords:** Scarabaeinae, Caatinga, semiarid

## Abstract

Dung beetle (Coleoptera: Scarabaeoidea: Scarabaeinae) activity is influenced by rainfall seasonality. We hypothesized that rainfall might also play a major role in regulating the community structure of this group. In this study, we describe seasonal changes in the richness, composition, and structure of the Scarabaeinae community in a Brazilian tropical dry forest. A fragment of arboreal Caatinga was sampled using baited pitfall traps during the early dry season (EDS), late dry season (LDS), early wet season (EWS), and middle wet season (MWS). We compared the dung beetle community in each season in relationship to species richness, rank-dominance, curves, and composition. We collected 1352 Scarabaeinae individuals , belonging to 15 species. *Dichotomius* aff. *laevicollis* Felsche (Coleoptera: Scarabaeidae) was the dominant species, representing 73.89% of the individuals. There were no seasonal changes in the rank dominance curves; all had a single dominant species and a few species with low abundance, typical for arid areas. Estimated richness was highest in MWS, followed by EWS. Dry-season samples (EDS and LDS) had lower richness, with no significant difference between the dry seasons. Although species richness increased as the habitat became wetter, the difference between the wet and dry seasons was small, which differs completely from the findings of other studies in Neotropical dry forests, where almost all species cease activities in the dry season. Species composition changes were found in non-metric multidimensional scaling and sustained by analysis of similarity. All the seasons had pairwise differences in composition, with the exception of EDS and MWS, which indicates that the dung beetle community in this fragment requires more than three months of drought to trigger changes in species composition; this is probably due to small changes in the forest canopy. There was no difference in composition between EDS and MWS. As in other tropical dry forests, although to a lesser extent, the dung beetle community of this fragment responded to rainfall seasonality with changes in species composition and reduced species richness. Such responses, even to this lesser extent, may occur because of small changes in tree cover and minor microclimate changes.

## Introduction

Dung beetles (Coleoptera: Scarabaeoidea: Scarabaeinae) are detritivores and mainly consume mammal dung, although some may eat dung from other animals and even from such distinct sources as carrion, fungi, and rotten fruit ( Halffter and Matthews 1966 ). Their feeding behavior is important for the ecosystem because it improves the quality of the soil (nutrient cycling, improved aeration, and water permeability), increases the secondary dispersal of seeds found in the dung, and reduces the populations of flies and worms in agroecosystems (reviewed in Nichols et al. 2008 ). Over the past 20 years, dung beetles have been used increasingly as bioindicators because of their accurate response to environment modifications; they are also an easily-sampled (at low costs) and well-known taxon ( Favila and Halffter 1997 , Gardner et al. 2008b ).

This group has received a great deal of attention in ecological studies conducted in tropical wet forests in the Neotropical region ( Gardner et al. 2008a , Louzada et al. 2010 ), but the same is not the case for tropical dry forests ( Andresen 2005 ). Tropical dry forests comprise vegetations that experiences six months of drought and are strongly seasonal ( Murphy and Lugo 1986 ); Brazilian Caatinga falls within this formation.

The Caatinga biome is composed of xerophytic and deciduous vegetation that extends for ≈735,000 km ^2^ over the northeast region of Brazil ( Leal et al. 2005 ). It experiences eight months of drought, with four mounths of rain usually concentrated in the winter, an annual precipitation ranging from 700 to 1,200 mm (in the most humid areas); average annual temperature varies between 26 and 28°C ( Cardoso and Queiroz 2010 ). This biome is highly endangered because of human activity ( Kirmse et al. 1987 , Li and Zhang 2000).

Rainfall seasonality is an important factor in dry forest communities ( Murphy and Lugo 1986 ) and, because dung beetle activity in tropical regions is often synchronized or maximized in line with rainfall ( Hanski and Cambefort 1991 ), it is expected that rainfall plays a major role in year-round species abundance. One pattern that has clearly emerged from the few studies of dung beetles in tropical dry forests is the drastic change in community composition from the wet season to the dry season, with a sharp reduction in species richness and abundance during the latter ( Andresen 2005 , Hernández 2007 . Neves et al. 2010 , Liberal et al. 2011).

Water deficit imposes changes on the dung beetle community in two ways. Because dung beetles mainly consume the nutrients present in dung water content ( Aschenborn et al. 1989 ), loss of water reduces dung quality. Furthermore, drought reduces the abundance of mammals in the environment, thereby reducing the availability of resources ( Hernández 2005 ). A dung beetle community also is directly influenced by high insolation and temperatures, factors that the beetles must adapt to during an invasion of arid areas ( Halffter and Matthews 1966 ). Most dung beetles of the Neotropical region cannot easily tolerate these conditions because of the negative effect of open area microclimatic condi-conditions (e.g., temperature) on adult and larvae survival ( Klein 1989 ). The aim of this study, therefore, was to test the hypothesis that environmental seasonality drives changes in the species richness and composition of the Scarabaeinae community in a Brazilian tropical dry forest.

## Materials and Methods

The study area was a 15,000 m fragment of Arboreal Caatinga (12°53'S 39°51'W), located in the eastern section of the Caatinga biome. The entire area is threatened by human activity and is classified as a high priority for conservation and in need of restoration (Velloso et al. 2002). It is located at the top of a 600 m slope and, as with most Caatinga, has a canopy height of 3-4 m with dense and shrubby understory vegetation. This fragment falls within a region with many inselbergs, surrounded by a matrix of pastures and shrubby Caatinga used for livestock activities. During the sampling period, the average temperature and precipitation were 24.8°C and 152.65 mm, respectively ( Ingá 2011 ).

### Sampling

**Figure 1.**
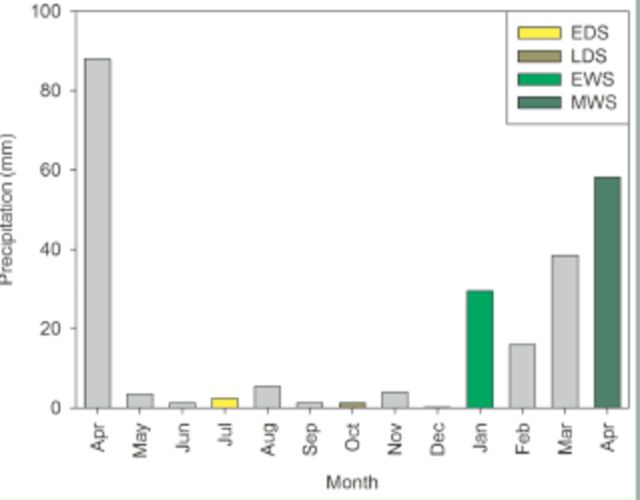
Rainfall precipitation (mm) during 10 and 11 April. Early dry season (EDS) corresponds to July 2010, late dry season (LDS) to October 2010, early wet season (EWS) to January 2011, and middle wet season (MWS) to April 2011.

Dung beetles were sampled using baited pitfall traps made from a plastic bin with a diameter of 14 cm and a depth of 10 cm. To avoid an edge effect, we set up a grid in the middle of the fragment at a minimum distance of 30 m from the border. The grid was arranged in rows of 4 × 4, evenly interspersed at a distance of 25 m, providing 16 sampling points. Each sampling point had four pitfalls placed at a distance of 2 m, which were baited with 25 mL of human dung, carrion (bovine spleen decayed for two days), cow dung, and rotten bananas, to maximize the sampling of specimens due to different feeding habits. The traps remained in the field for 48 h, after which they were emptied. This protocol was applied in the early dry season (EDS), late dry season (LDS), early wet season (EWS), and middle wet season (MWS), in July and October 2010 and January and April 2011, respectively ( Fig. 1 ). Dung beetles were identified using a key for genera ( Vaz-de-Mello et al. 2011 ) and making comparisons with specimens of identified species in the Johan Becker entomological collection of the Museum of Zoology of the State University of Feira de Santana (Museu de Zoologia da UEFS: MZFS), and by consulting specialists.

### Statistical analysis

Individual-based rarefaction curves were constructed to compare observed species richness (Sobs) between seasons, avoiding the effects of individual abundance on results, because of the tendency for more individuals to be active during rainy seasons. We also calculated the nonparametric richness estimator Jackknife 2 with 100 randomizations to evaluate the completeness of the sampling effort and compared expected species richness between seasons when rarefaction curves did not reach an asymptote. These analyses were conducted using the EstimateS 8.2 package ( Colwell 2009 ).

The dung beetle community between seasons also was compared through a visual inspection of rank-dominance curves with species relative abundance. Variations in species composition between seasons were tested using non-metric multidimensional scaling (NMDS) with 30 restarts, and abundance data were square root transformed before creating a resemblance matrix creation, using the Bray–Curtis dissimilarity index. The species composition patterns found using NMDS were validated through a one-way ANOSIM with 50,000 permutations, using Primer 6.0 ( Clarke 1993 ) software.

## Results

**Figure 2.**
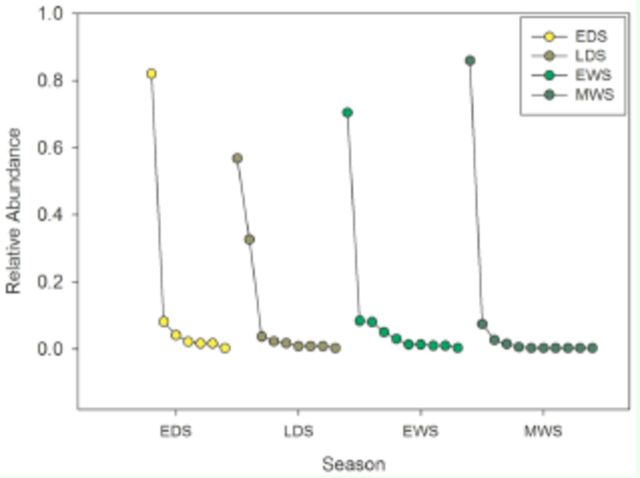
Rank species abundance plots for dung beetle assemblages at four different moments: early dry season (EDS), late dry season (LDS), early wet season (EWS) and middle wet season (MWS).

A total of 1,352 Scarabaeinae individuals were collected, belonging to 15 species and 8 genera ( Table 1 ). *Dichotomius* aff. *laevicollis* Felsche (Coleoptera: Scarabaeidae) was the most abundant species, representing 73.89% of total individuals, followed by *Canthon rutilans* Laporte with 14.35%. Other species were each responsible for less than 5% of relative abundance. Species typical of Brazilian tropical dry forests, such as *Deltochilum verruciferum* Felsche and *Coprophanaeus pertyi* Olsoufieff (Caatinga) were recorded. However, species previously only recorded in Atlantic forest also were found, such as *Canton stage* Pereira and *Canthidium* sp 2. The rank dominance curves for the four sampling seasons were very similar ( Fig. 2 ), although there was an increase in the abundance of *Canthon rutilans* in the late dry season.

**Table 1.**
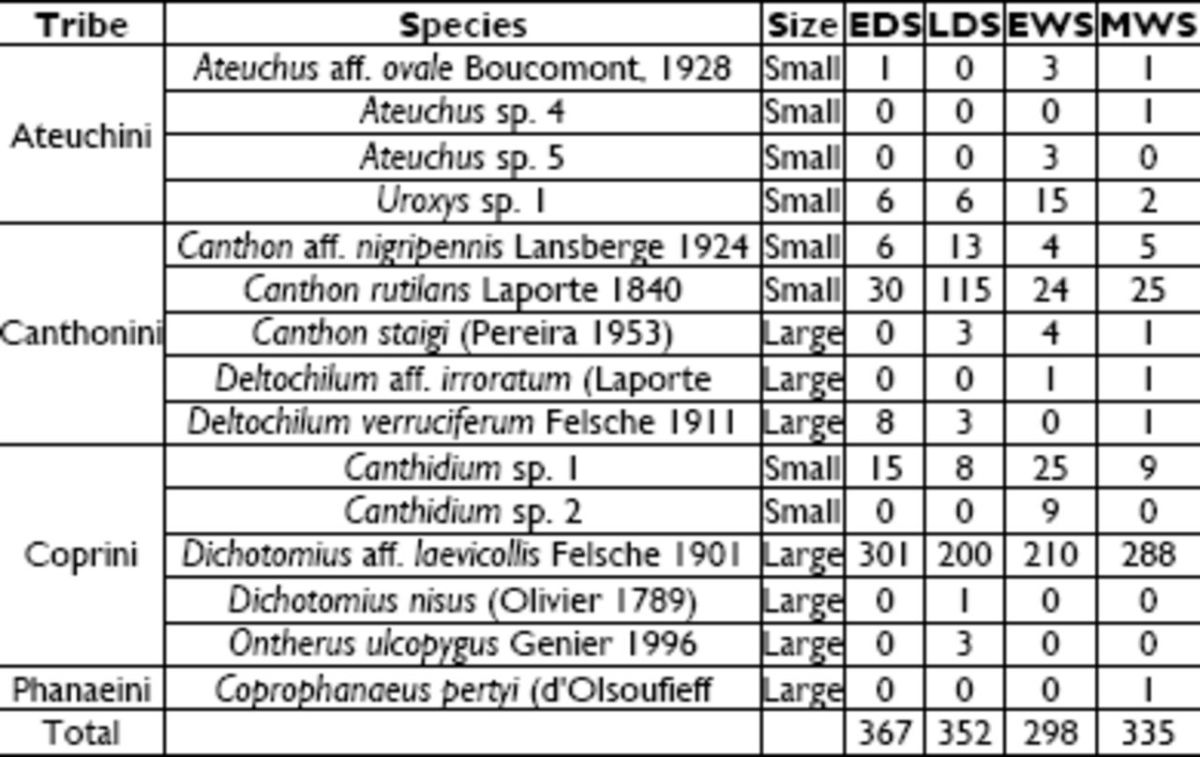
Rainfall precipitation (mm) during 10 and 11 April. Early dry season (EDS) corresponds to July 2010, late dry season (LDS) to October 2010, early wet season (EWS) to January 2011, and middle wet season (MWS) to April 2011.

Species of dung beetle with abundance and size-class (small ≤ 1.0 cm, large > 1.0 cm), sampled from arboreal Caatinga during four sampling periods: early dry season (EDS), late dry season (LDS), early wet season (EWS), and middle wet season (MWS).

**Figure 3.**
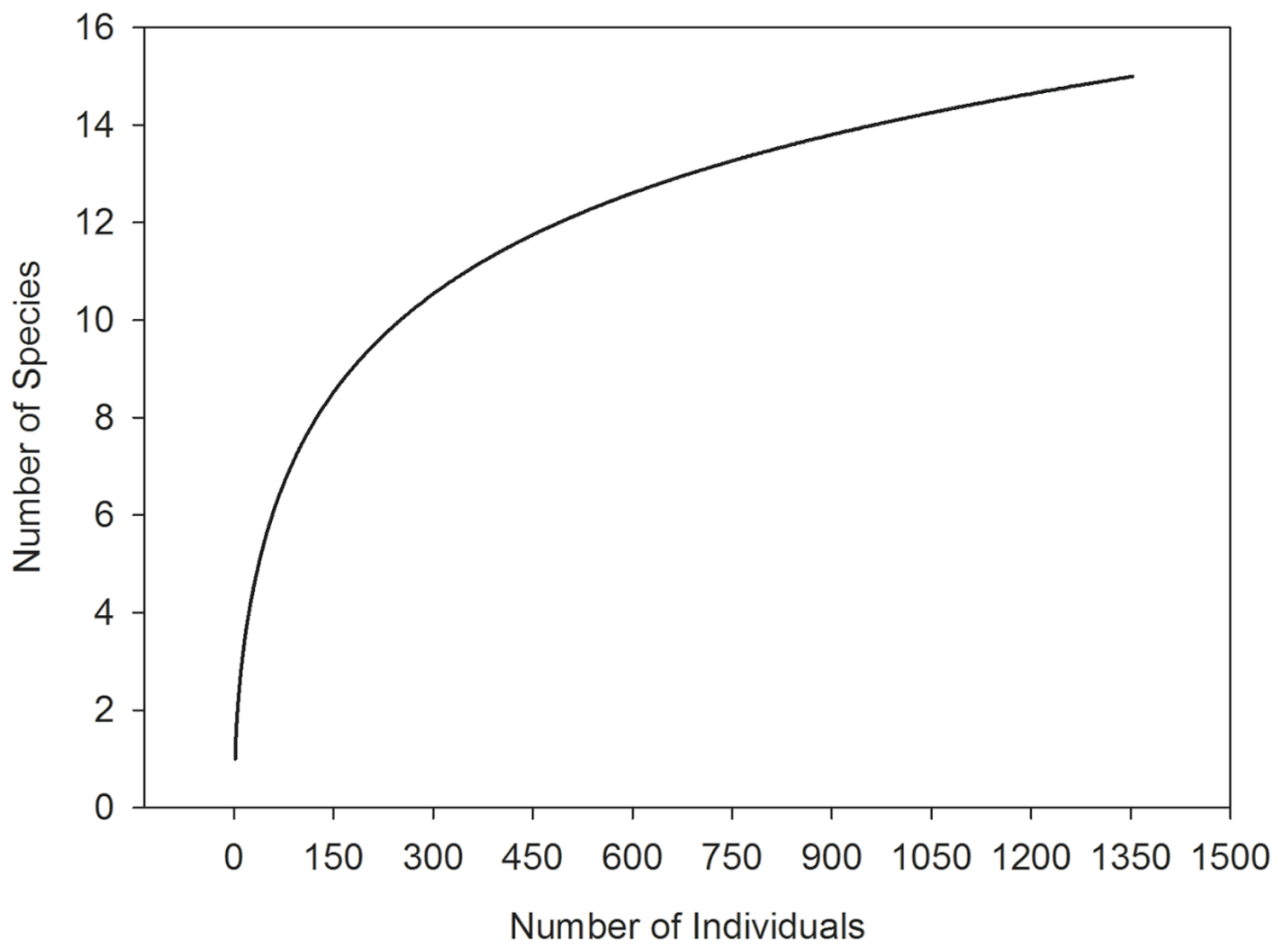
Individual-based rarefaction for the community.

**Figure 4.**
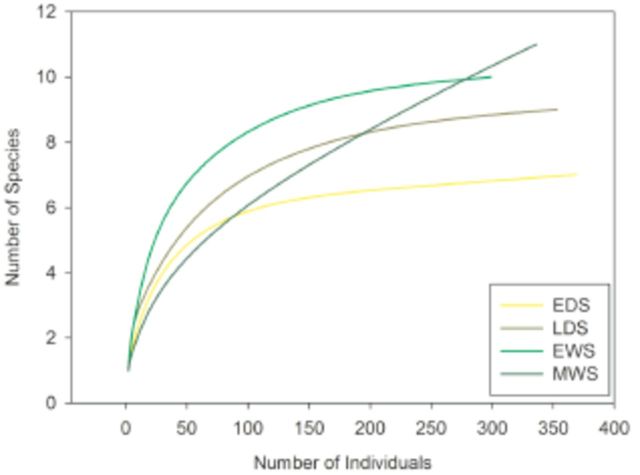
Individual-based rarefaction comparing the early dry season (EDS), late dry season (LDS), early wet season (EWS), and middle wet season (MWS).

The rarefaction curve did not reach an asymptote ( Fig. 3 ), and the total richness estimated by Jackknife 2 was 20, indicating sampling effort completeness of 75%. The MWS may interfere with curve behavior for the entire area because the percentage of species richness sampled for EDS, LDS, and EWS ranged from 77.86 to 83.4%, and in the MWS, only 50.09% of the estimated species were sampled. Observed species richness by season was 7 for EDS, 9 for LDS, 10 for EWS, and 11 for MWS. A comparison of rarefaction curves between seasons ( Fig. 4 ) reveals statistical differences that indicate small but significant changes in species richness (rarefaction curves results: 6.8 ± 0.35 (EDS); 8.84 ± 0.29 (LDS); 10 ± 0.01 (EWS); 10.33 ± 4.82 (MWS)). Although MWS had the highest richness, this was not different from the other seasons, because the richness curve did not reach an asymptotic value, meaning that any comparisons of observed richness remain inconclusive.

Comparison between estimated richness of Jackknife 2 shows that MWS had the highest richness, followed by EWS and then the dry seasons (EDS and LDS), which had lower richness, although there were no differences between the dry seasons (estimated richness results: 8.4 ± 0,14; 9.98 ± 0.19; 11.99 ± 0.01; 20.44 ± 0.30, respectively). This higher Jackknife 2 estimation value was related to an increase in the proportion of singletons and doubletons, which may be associated with the beginning of species emerging as a consequence of rain.

**Figure 5.**
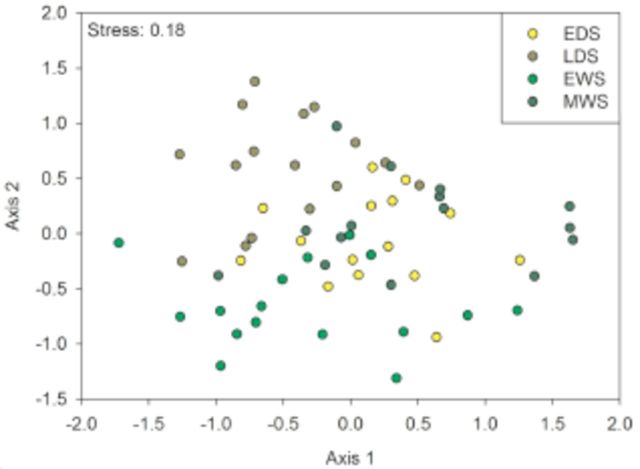
Non-metric multidimensional scaling (NMDS) ordination, based on a distance matrix using Bray-Curtis dissimilarity, of the early dry season (EDS), late dry season (LDS), early wet season (EWS), and middle wet season.

Composition differences in the community structure were found via NMDS ( Fig. 5 ), with clear differences between the early wet and other seasons, which is sustained by ANOSIM ( *P* < 0.05). All seasons had pairwise differences in species composition (R >0.2), ranging from 0.214 between EDS and EWS to 0.305 between EDS and LDS; the only exception was EDS, which did not differ from MWS.

## Discussion

The dung beetle species richness found in this study is low compared with those found in tropical rainforest communities ( Klein 1989 [55 spp.], Andresen 2002 [59 spp.], Hernández and Vaz de Mello 2009 [39 spp.], Lopes et al. 2011 [27 spp.]), but similar to the richness found in other Caatingas ( Hernández 2005 [20 spp.], Hernández 2007 [20 spp.], Lopes et al. 2006 [16 spp.]) and other tropical dry forests ( Escobar 1997 [22 spp.]; Andresen 2005 [15 spp.], Andresen 2008 [15 spp.], Liberal et al. 2011 [13 spp.]), all in Neotropical regions. Other biodiversity studies in tropical dry forests have manifested different numbers of species ( Lopes and Louzada 2005 [4 spp.], Neves et al. 2010 [32 spp.]). Even given the sampling differences (number of pitfalls, type and number of baits used, number of areas sampled, and fragment size), our study presents with high richness. This is surprising because of the small size of the fragment, the fact that it does not fall within a conservation area, and it is surrounded by areas used for livestock breeding.

*Dichotomius nisus* Olivier and *Ontherus ulcopygus* Génier were exclusively collected in LDS; the former is typical of open areas ( Louzada et al. 2007 ) and may use the fragment as a refuge from the driest conditions. Two species were caught exclusively in EWS *(Canthidium* sp 2 and *Ateuchus* sp 5) and were found in banana traps, which may be related to the beginning of fruit availability in the environment; two species were caught exclusively in MWS *(Coprophanaeus pertyi* Olsoufieff and *Ateuchus* sp 4).

There was no modification in the format of rank dominance curves as a consequence of the change from the dry to wet season and only a small increase in species richness in wetter conditions. The general shape of all the curves demonstrates a pattern of communities with the strong dominance of one species. Dung beetle communities with such patterns are common in disturbed environments and areas with a marked dry season ( Andresen 2005 ), and this may also be regarded as an indication either that the majority of Neotropical dung beetle species do not tolerate the adverse environmental conditions of tropical dry forests or that their population sizes are greatly reduced because of the small size of mammal populations.

It is interesting to note, however, that the dominant species in our study belongs to a group of species typical of rainforests ( Silva et al. 2010 ), despite being recorded in Caatinga ( Liberal et al. 2011 ). Because the dung beetles of the Neotropical region have an evolutionary history closely related to tropical rain forests and are sensitive to the reduction in tree cover and its consequences (insolation, temperature and rain) ( Halffter and Arellano 2002 ), one would not expect to find this species in a tropical dry forest, or at the least not as the dominant species.

Denser and continuous forest canopies allow more stable microclimatic conditions at ground level, so that open areas, such as pastures, have microclimatic characteristics different, in both mean values and variability, from those in forest areas ( Klein 1989 ). It is therefore expected that more closed Caatinga formations would more closely resemble the latter rather than the former, thereby enhancing occupation by rainforest species. This has been observed in a Caatinga forest located on the slope of a hill and in a “brejo nordestino”, which is a rainforest relict surrounded by more arid areas. Such locations may have characteristics more favorable to dung beetles, with higher richness [28 spp.] compared with other Caatinga dung beetle communities and with records of dung beetle species in Atlantic forests ( Silva et al. 2007 ). Given that the area sampled in this study is located on the southwestern border of the Caatinga biome near other more humid biomes, such as Atlantic forest, its occupation by rainforest species is only possible if the arboreal structure of this and other arboreal Caatingas function as a climatic and vegetation refuge from the dryer conditions of nearby shrubby Caatingas and pastures for mammals and Scarabaeinae.

Although there was an increase in estimated dung beetle richness as the habitat got wetter, the differences we found in estimated richness between the wet and dry seasons were small. This was radically different from other studies, which found reductions of more than 76% in species richness and where species with greater body lengths were more prone to cease activities in the dry season ( Janzen 1983 , Andresen 2005 , Neves et al. 2010 , Liberal et al. 2011 ), with the single exception of one study, which manifested more species in the dry season ( Escobar 1997 ).

The low intensity of changes in tree coverage that occurred during our study probably resulted in minor microclimatic changes in solar radiation, humidity, and temperature across seasons; had these changes been more intense they would have had a negative effect on dung beetle species richness ( Halffter and Edmonds 1982 , Klein 1989 , Lobo et al. 1998 ). Furthermore, the soil in this fragment did not manifest any signs of compaction and dryness (personal observations) that could hinder dung beetle excavation ( Janzen 1983 ). These aspects may help explain the slight changes in species richness. This region has inselbergs and associated Caatinga formations that tend to be more humid and contain leaf litter ( Santos et al. 1999 ), thus allowing more species and individuals to be active during the dry seasons.

On the other hand, changes in dung beetle composition elsewhere have been related to variations in tree coverage ( Halffter and Arellano 2002 ), so that small changes in the dung beetle community are more detectable in species composition than in species richness. This difference was detectable in all comparisons between sampling seasons, with the exception of pairwise comparisons between EDS and MWS. Because there were no differences between these, probably because of only small changes in the canopy of this plant formation, it is possible that more than three months of drought are required to trigger changes in species composition from the end of the wet season to the beginning of the dry season. These are rather speculative results, however, because EDS was not sampled following MWS.

Caatinga possesses a plant community that is typically xerophytic ( Prado 2008 ), with various strategies for saving water, such as succulence and deciduousness. Rain seasonality regulates Caatingas’ phenology, but irregularity of rainfall may also affect the plant community’s response. If the rainfall during the rainy season is higher than normal, changes in seed production ( Silva et al. 2013 ) and loss of leaves (and the resulting maintenance of canopy continuity) may affect the fauna. The effect of small changes in microclimatic conditions may allow the survival of species that would otherwise disappear and the maintenance of a certain rainforest species as its dominant species. The small but significant rise in species richness as the wet season begins is expected and may be the result of reproductive cycles, which are triggered by the detection of higher humidity in the soil.

Although to a lesser extent than in other tropical dry forests, the dung beetle community in this fragment responded to rainfall in relationship to species richness and composition. Given that this fragment is located close to the frontier between Caatinga and Atlantic forest domains, further studies focusing on faunistic composition similarity and the migration of species between these two formations could answer some of the questions presented here and also the effect of distance between Caatinga and Atlantic Forest on species richness and composition.

## References

[R1] Andresen E . 2002 . Dung beetles in a Central Amazonian rainforest and their ecological role as secondary seed dispersers . *Ecol. Entomol.* 27 : 257-270.

[R2] Andresen E . 2005 . Effects of season and vegetation type on community organization of dung beetles in a tropical dry forest . *Biotropica* 37 : 291-300.

[R3] Andresen E . 2008 . Dung beetle assemblages in primary forest and disturbed habitats in a tropical dry forest landscape in western Mexico . J. Insect Conserv. 12 : 639-650.

[R4] Aschenborn H. H. Loughnan M. L. Edwards P. B. . 1989 . A simple assay to determine the nutritional suitability of cattle dung for coprophagous beetles . Entomol. Exp. Appl. 53 : 73-79

[R5] Cardoso D.B.O.S. Queiroz L. P. . 2010 . Caatinga no contexto de uma Metacomunidade: Evidências da Biogeografia, Padrões Filogenéticos e Abundância de Espécies em Leguminosas, pp. 241-260. *In* C.C.J.B. Carvalho and E.A.B. Almeida (Org.). *Biogeografia da América do Sul: padrões e processos.* Roca. (in Portuguese)

[R6] Clarke K. R . 1993 . Nonparametric multivariate analyses of changes in community structure . Austral Ecol. 18 : 117-143

[R7] Colwell R. K . 2009 . EstimateS: Statistical estimation of species richness and shared species from samples. Version 8.2. User's Guide and application published at: http://viceroy.eeb.uconn.edu/estimates/

[R8] Escobar F . 1997 . Estudio de la Comunidad de Coleopteros Coprofagos (Scarabaeidae) en un Remanente de Bosque Seco al Norte del Tolima, Colombia . Caldasia 19 : 419-430. (in Spanish)

[R9] Favila M. E. Halffter G. . 1997 . The use of indicator groups for measuring biodiversity as related to community structure and function . Acta Zool. Mex. 72 : 1-25.

[R10] Gardner T.A. Barlow J. Araujo I. S. Ávila-Pires T. C. Bonaldo A. B. Costa J. E. Esposito M. C. Ferreira L. V. Hawes J. Hernández M. I. M. Hoogmoed M. S. Leite R. N. Lo-Man-Hung N. F. Malcolm J. R. Martins M. B. Mestre L. A. M. Miranda-Santos R. Overal W. L. Parry L. Peters S. L. Ribeiro-Junior M. A. da Silva M. N. F. Motta C. S. Peres C. A. . 2008a . The cost-effectiveness of biodiversity surveys in tropical forests. *Ecol. Lett.* 11 : 139-150. 10.1111/j.1461-0248.2007.01133.x 18031554

[R11] Gardner T. A. Hernández M. I. M. Barlow J. Peres C. A. . 2008b . Understanding the biodiversity consequences of habitat change: the value of secondary and plantation forests for neotropical dung beetles. *J. Appl. Ecol.* 45 : 883-893.

[R12] Halffter G Arellano L. . 2002 . Response of dung beetle diversity to human-induced changes in a tropical landscape. *Biotropica* 34 : 144-154.

[R13] Halffter G Edmonds W. D. 1982 . *The nesting behavior of dung beetles (Scarabaeinae). an ecological and evolutive approach.* Man and the Biosphere Program UNESCO, México D.F.

[R14] Halffter G Matthews E. . 1966 . Natural history of dung beetles of the subfamily Scarabaeinae (Coleoptera, Scarabaeidae). *Fol. Entomol. Mex.* 12- 14 : 1-312.

[R15] Hanski I Cambefort Y. . 1991 . *Dung beetle ecology.* Princeton University Press, Princeton, NJ.

[R16] Hernández M. I. M. 2005 . Artrópodes: besouros Scarabaeidae (Coleoptera) do Curimataú, Paraíba, pp. 369-380. *In* F. S. Araújo, M. J. N. Rodal, and M. R. V. Barbosa (eds.). *Análise das variações da biodiversidade do bioma Caatinga para suporte a estratégias regionais de conservação.* Ministério do Meio Ambiente, Brasília . (in Portuguese)

[R17] Hernández M. I. M. 2007 . Besouros escarabeíneos (Coleoptera: Scarabaeidae) da Caatinga paraibana, Brasil . *Oecol. Brasi.* 11 : 356-364. (in Portuguese)

[R18] Hernández M. I. M. Vaz-de-Mello F. Z. 2009 . Seasonal and spatial species richness variation of dung beetle (Coleoptera, Scarabaeidae s. str.) in the Atlantic Forest of southeastern Brazil . *Rev. Brasil. Entomol.* 53 : 607-613.

[R19] Ingá M . 2011 . *Pluviometria diária de Milagres.* Available online: http://www.inema.ba.gov.br

[R20] Janzen D. H . 1983 . Seasonal change in abundance of large nocturnal dung beetles (Scarabaeidae) in a Costa Rican deciduous forest and adjacent horse pasture . Oikos 41 : 274-283

[R21] Kirmse R. D. Provenza F. D. Malechek J. C. 1987 . Clearcutting Brazilian caatinga: assessment of a traditional forest grazing management practice . Agrofor. Syst. 5 : 429-441.

[R22] Klein B. C . 1989 . Effects of forest fragmentation on dung and carrion beetle communities in central Amazonia . Ecology 70 : 1715-1725

[R23] Leal I. R. Silva J. M. C. Tabarelli M. Lacher T. E. 2005 . Changing the course of biodiversity conservation in the Caatinga of northeastern Brazil . Conserv. Biol. 19 : 701-706.

[R24] Li L. E. Zhang. W. L. 2000 . Native vegetation and its ecosystem current situation in Brazil . J. For. Res. 11 : 140-144

[R25] Liberal C. N. Isidro de Farias M. Meiado M. V. Filgueiras B. K. C. Iannuzzi L. 2011 . How habitat change and rainfall affect dung beetle diversity in Caatinga, a Brazilian semiarid ecosystem . J. Insect Sci. 11 :114 Available online: www.insectscience.org/11.114 10.1673/031.011.11401 PMC3281362 22224924

[R26] Lobo J. M. Lumaret J. P. Jay-Robert P. 1998 . Sampling dung beetles in the French Mediterranean area: effects of abiotic factors and farm practices . Pedobiologia 42 : 252 – 266 .

[R27] Lopes J. V. Korasaki L. L. Catelli V. V. Marçal M. Nunes M.P.B.P. 2011 . A comparison of dung beetle assemblage structure (Coleoptera: Scarabaeidae: Scarabaeinae) between an Atlantic forest fragment and adjacent abandoned pasture in Paraná, Brazil . Zoologia (Curitiba, Impresso) 28 : 72 – 79 .

[R28] Lopes P. P. Louzada. J. N. C. 2005 . Besouros (Scarabaeidae e Histeridae), pp. 284‒298. *In* F. A. Juncá, L. Funch, and W. Rocha (eds.). *Biodiversidade e Conservação da Chapada Diamantina* . Ministério do Meio Ambiente, Brasília. (in Portuguese)

[R29] Lopes P. P. Louzada J. N. C. Vaz-de-Mello F. Z. 2006 . Organization of dung beetle communities (Coleoptera, Scarabaeidae) in areas of vegetation re-establishment in Feira de Santana, Bahia, Brazil . Sitientibus Sér. Ciên. Biol . 6 : 261 – 266 .

[R30] Louzada J. N. C. Lopes F. S. Vaz-de-Mello F. Z. . 2007 . Structure and composition of a dung beetle community (Coleoptera, Scarabaeinae) in a small forest patch from Brazilian Pantanal . Rev. Brasil. Zooci. 9 : 199 – 203

[R31] Louzada J Gardner T. A. Peres C. A. Barlow J. 2010 . A multi-taxa assessment of nestedness patterns across a multiple-use Amazonian forest landscape . Biol. Conserv . 143 : 1102 – 1109

[R32] Murphy P. G. Lugo E. . 1986 . Ecology of tropical dry forest . Annu. Rev. Ecol. Syst . 17 : 67 – 88 .

[R33] Neves F. S. Oliveira V. H. F. Vaz-de-Mello F. Z Louzada J. Sanchez-Azofeifa A. Fernandes G. W. . 2010 . Successional and seasonal changes in a community of dung beetles (Coleoptera: Scarabaeinae) in a Brazilian tropical dry forest . Nat. Conserv. 8 : 160-164.

[R34] Nichols E Spector S. Louzada J Larsen T. Amezquita S. Favila M. . 2008 . Ecological functions and ecosystem services provided by Scarabaeinae dung beetles . Biol. Conserv . 141 : 1461-1474

[R35] Prado D. E . 2008 . As Caatingas da América do Sul, pp. 3-73. *In* I. R. Leal, M. Taberelli, and J. M. C. Silva (eds). *Ecologia e conservação da Caatinga.* Recife, Ed. Universitária-UFPE. (in Portuguese)

[R36] Santos G. M. M. Delabie J. H. C. Resende J. J. 1999 . Caracterização da mirmecofauna (Hymenoptera-Formicidae) associada à vegetação periférica de Inselbergs (Caatinga arbórea estacional semidecídua) em Itatim, Bahia, Brasil. *Sitien. Sér. Ciê. Biol.* 20 : 33-43 (in Portuguese)

[R37] Silva F. A. B. Hernández M. I. M. Ide S. Moura R. C. . 2007 . Comunidade de escarabeíneos (Coleoptera, Scarabaeidae) copro-necrófagos da região de Brejo Novo, Caruaru, Pernambuco, Brasil . Rev. Brasil. Entomol. 51 : 228-233. (in Portuguese).

[R38] Silva F.A. B. Costa C. M. Q. Moura R.C Farias A. I. . 2010 . Study of the dung beetle (Coleoptera: Scarabaeidae) community at two sites: Atlantic forest and clearcut, Pernambuco, Brazil . Environ. Entomol. 39 : 359-367. 10.1603/EN09180 20388264

[R39] Silva K. A. Santos D. M. Santos J.M.F.F. Albuquerque U. P. Ferraz E. M. N. Araújo E. L. . 2013 . Spatiotemporal variation in a seed bank of a semiarid region in northeastern Brazil . Acta Oecol . 46 : 25 – 32 .

[R40] Vaz-de-Mello F. Z. Edmonds W. D. Ocampo F. C. Schoolmeesters P. 2011 . A multilingual key to the genera and subgenera of the subfamily Scarabaeinae of the New World (Coleoptera: Scarabaeidae) . Zootaxa 2854 : 1 – 73 .

[R41] Velloso A. L. Sampaio E.V.S.B. Pareyn F. G. C. . 2002 . *Ecorregiões propostas para o Bioma Caatinga* . Associação Plantas do Nordeste, Instituto de Conservação Ambiental, The Nature Conservancy do Brasil, Recife, Brasil .

